# The impact of patient age on the oncological prognosis of oral squamous cell carcinoma

**DOI:** 10.1007/s10006-026-01546-4

**Published:** 2026-03-11

**Authors:** Friedrich Mrosk, Victoria Vertic, Maximilian Richter, Lukas Mödl, Erin Sprünken, Jan Oliver Voss, Anna Sofroniou, Christian Doll, Carsten Rendenbach, Max Heiland, Steffen Koerdt

**Affiliations:** 1 Department of Oral and Maxillofacial Surgery, Charité – Universitätsmedizin Berlin, Corporate Member of Freie Universität Berlin and Humboldt-Universität zu Berlin, Augustenburger Platz 1, Berlin, 13353 Germany; 2 Institute of Biometry and Clinical Epidemiolgy, Charité – Universitätsmedizin Berlin, Corporate Member of Freie Universität Berlin and Humboldt-Universität zu Berlin, Charitéplatz Platz 1, Berlin, 10117 Germany

**Keywords:** Oral squamous cell carcinoma, Prognosis, Age, Risk factors, Survival

## Abstract

**Objectives:**

Epidemiological data show that while age-standardized mortality rates of oral squamous cell carcinoma (OSCC) have slightly declined, the absolute number of deaths continues to rise, with a concerning increase among younger patients and persistently high mortality in the elderly. The aim of this study was to assess the influence of age on oncological prognosis and to classify it within a multivariable model alongside established histopathological risk factors.

**Methods:**

In this retrospective single-center study, patients surgically treated for OSCC between 2012 and 2023 were included according to predefined eligibility criteria and stratified into three age groups (< 50, 50–69, ≥ 70 years). Primary endpoints were overall survival (OS) and disease-free survival (DFS). Prognostic factors were further evaluated with multivariable Cox regression models and competing-risk analyses, with age assessed both as a categorical and continuous variable using restricted cubic splines.

**Results:**

In 525 included patients (mean age 63.5 years), age distribution was multimodal with three peaks in concordance with the predefined groups. Local recurrence, distant metastasis, and secondary cervical lymph node metastasis (CLNM) were weakly correlated overall, with a stronger association between CLNM and distant metastasis in younger patients. Age significantly predicted overall survival (HR per 10 years: 1.56, 95% CI: 1.29–1.90), but showed no independent association with disease-free survival.

**Conclusions:**

Age presented with a multimodal distribution with distinct clinical patterns across the subgroups and was independently associated with worse OS. Nevertheless, no association was observed for DFS, suggesting that age-related differences in mortality may be influenced by non-oncologic factors. While correlations between recurrence events were generally weak, younger patients displayed a stronger link between regional and distant metastasis, emphasizing the importance of age-related follow-up.

## Introduction

Oral squamous cell carcinoma (OSCC) is associated with considerable morbidity and mortality, with patient survival remaining unsatisfactory despite advances in diagnosis and treatment over the last decades. According to GLOBOCAN 2020, lip and oral cavity cancers accounted for an estimated 177,757 deaths, with an age-standardized mortality rate of 2.0 per 100,000, markedly higher in men than in women and with the highest burden in South Asia and parts of Eastern Europe [1]. More recent GLOBOCAN 2022 data indicate a global lifetime risk of death from oral cancers of approximately 0.5%, with men facing more than twice the risk compared to women, and mortality risk remaining substantial even at older ages [2]. Complementary analyses from the Global Burden of Disease suggest that while age-standardized mortality rates have slightly declined over recent decades, the absolute number of deaths continues to increase [3]. This is likely due to the aging overall population. Furthermore, patients under 44 years, and especially those aged 20–24, showed the steepest increases over the past three decades [3]. These patterns suggest a shifting prognostic relevance of age, with younger patients emerging as an increasingly affected subgroup. Established risk factors for the development of head and neck cancer in general are smoking, alcohol consumption and HPV [4, 5]. Here, recent studies have shown that the risk increases in relation to the age of starting smoking, suggesting that the duration and cumulative risk plays a major role [6]. Nevertheless, the oncological prognosis for OSCC is also dependent on several histopathological risk factors, such as the disease stage, presence and extent of lymph node metastasis, the infiltrative pattern or treatment adherence [7–9]. There is evidence suggesting that the relevance and impact of these factors may vary according to patient age [10, 11].

Although increasing age has consistently been associated with inferior overall survival in head and neck cancer, several studies have demonstrated that this association is substantially attenuated after adjustment for comorbidity burden and frailty indices. These findings raise the question whether chronological age reflects intrinsic tumor biology or rather accumulated health deficits and competing mortality risks [12–14]. Few studies have explicitly attempted to disentangle these effects within multivariable models incorporating detailed histopathological parameters. Therefore, the aim of this study was not to assume biological differences a priori, but to evaluate whether age retains independent prognostic relevance after adjustment for established clinicopathological risk factors.

## Methods

### Study design

In this retrospective observational single-center study, all patients who were surgically treated for histologically confirmed OSCC between 01/2012 and 12/2023 at the Department of Oral and Maxillofacial Surgery, Charité – Universitätsmedizin Berlin, were assessed for inclusion. Further inclusion criteria were primary treatment according to the current clinical guideline recommendations. Exclusion criteria were: (1) non-adherence to guideline-recommended adjuvant therapy, (2) secondary or concomitant carcinoma, (3) history of previous radiation to the head and neck and (4) incomplete data. Patients were initially grouped into three predefined age-groups: <50 years (young), 50–69 years (middle-aged), and ≥ 70 years (elderly). Age was determined at the time of primary treatment. Primary endpoints of the study were the overall survival (OS) and disease-free survival (DFS). Investigated variables included epidemiological, clinical and histopathological parameters. The study was approved by the local ethics committee (EA2-077-20) and conducted in accordance with the principles of the Declaration of Helsinki.

### Statistical analysis

Continuous variables were expressed as mean ± standard deviation (SD) and compared between groups using analysis of variance (ANOVA). Categorical variables were reported as absolute counts and percentages, with group differences assessed by the chi-square test. The ECS/pN+ ratio was calculated as: number of patients with extracapsular spread (ECS) divided by the total number of patients with histologically confirmed lymph node metastasis (pN+), expressed as a percentage within each age group. Patients age was evaluated both as a continuous and as a categorical variable. To characterize the age distribution within the cohort, we planned to generate histograms with kernel density estimation. Visual inspection of the distributions was complemented by statistical testing for multimodality using Hartigan’s dip test, and Gaussian Mixture Modeling (GMM) was considered to estimate the potential number of latent age subgroups. OS and DFS were defined as the time in months from diagnosis to death (OS) or disease recurrence (DFS), respectively. Patients without an event at last follow-up were censored. Survival functions were estimated using the Kaplan–Meier method and compared between the predefined age groups by log-rank test, reporting 3-year survival rates with their respective 95% confidence intervals (95%CI). Multivariable Cox proportional hazards regression models were constructed to evaluate prognostic factors for OS and DFS. Before inclusion in the Cox regression models, the variable age was examined using restricted cubic spline functions to test for potential non-linear effects. Additionally, competing-risk analyses were performed to account for potential bias in standard DFS modeling. Cumulative incidence functions were estimated with death without recurrence treated as a competing event, and multivariable Fine–Gray regression models were fitted, reporting subdistribution hazard ratios (sHR). All statistical analyses were performed using R (version 4.5.1, R Foundation for Statistical Computing). A two-sided p-value < 0.05 was considered to indicate statistical significance.

## Results

### Baseline characteristics

**Fig. 1:**
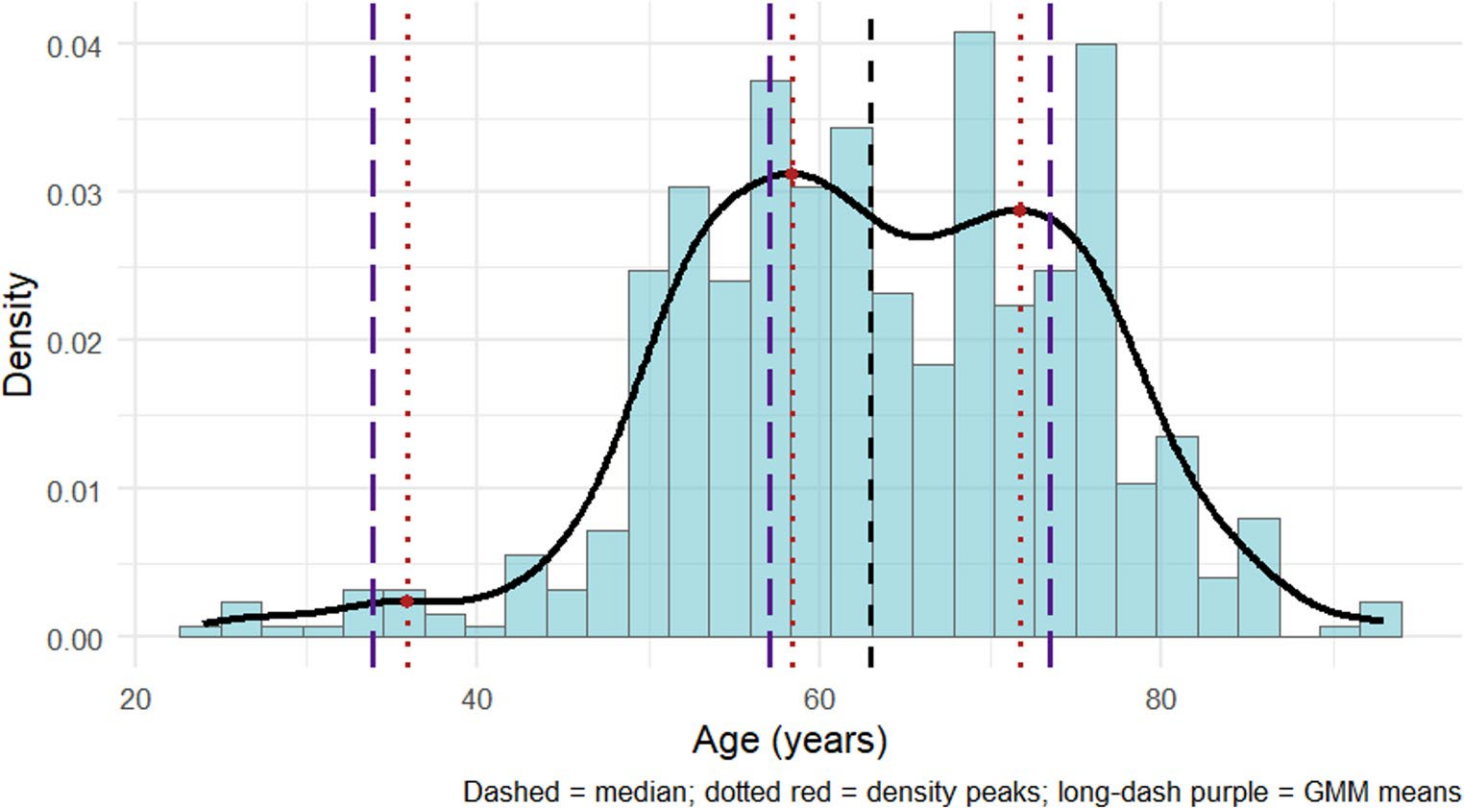
Multimodal age distribution of the study cohort, showing one major incidence peak in middle-aged patients, a secondary peak in elderly patients and a smaller increase in younger patients

Overall, 525 patients were included. The mean age of the whole cohort was 63.5 ± 11.7 years, ranging between 24 and 93 years. The distribution of patient age was multimodal (Hartigan’s dip test: D = 0.03, *p* < 0.01). Density analysis revealed three distinct peaks at 36, 58, and 72 years, while Gaussian Mixture Modeling confirmed that a three-component solution provided the best fit to the data. The estimated means of the three age subgroups were 33.8, 57.0, and 73.4 years, respectively. The final histogram with density overlay (Fig. 1) illustrates the multimodal age distribution with its three peaks. Neck management consisted of selective neck dissection (SND) in 413 (78.7%), modified radical neck dissection (MRND) in 125 (15.4%) and sentinel lymph node biopsy (SLNB) in 22 (4.2%) of cases. Adjuvant therapy was performed in 196 (37.3%) of cases and consisted of postoperative radiotherapy in 125 (63.8%) and postoperative chemoradiation in 71 (36.2%) cases. The baseline characteristics of the cohort are shown in Table 1. Younger patients presented with significantly more tongue OSCC while the proportion of upper jaw OSCC was the highest in the elderly group. Furthermore, there was a relatively higher proportion of floor of mouth carcinomas in middle-aged patients compared to the young and elderly group. Elderly patients tended to be more non-smoker, while the male sex was significantly more frequent in the middle-age group. There were no significant differences in relation to the T- and N-stage. Nevertheless, younger patients had a significantly higher ECS/pN+ ratio (62.5% vs. 35.3% vs. 25.5% per age group, respectively).

**Table 1:** Baseline characteristics of the study cohort according to the age groups

	Young (*n* = 45)	Middle-age (*n* = 305)	Elderly (*n* = 175)	*p*-Value
Age (mean ± SD)	41.2 ± 7.6	59.5 ± 5.7	76.1 ± 4.8	*< 0.01*
Smoking				< 0.01
Yes	30 (66.6)	218 (71.5)	93 (53.1)	
No	15 (33.3)	87 (28.5)	82 (46.9)	
Sex				< 0.01
Male	25 (55.6)	193 (63.3)	86 (49.1)	
Female	20 (44.4)	112 (36.7)	89 (50.9)	
Tumor location				< 0.01
Tongue	24 (53.3)	85 (27.9)	50 (28.6)	
Floor of mouth	6 (13.3)	108 (35.4)	38 (21.7)	
Lower jaw	10 (22.2)	56 (18.4)	45 (25.7)	
Buccal mucosa	2 (4.4)	22 (7.2)	14 (8.0)	
Upper jaw	2 (4.4)	12 (3.9)	20 (11.4)	
Multiple regions	1 (2.2)	22 (7.2)	8 (4.6)	
pT-stage				0.10
pT1	25 (55.6)	128 (43.8)	64 (36.6)	
pT2	11 (24.4)	98 (33.6)	62 (35.4)	
pT3	3 (6.7)	33 (11.3)	11 (6.3)	
pT4a	6 (13.3)	46 (15.7)	38 (21.7)	
pN-stage				0.36
pN0	37 (82.2)	220 (72.1)	128 (73.1)	
pN+	8 (17.8)	85 (27.9)	47 (26.9)	
Extracapsular spread				0.47
Yes	5 (11.1)	30 (9.8)	12 (6.9)	
No	40 (88.9)	275 (90.2)	163 (93.1)	
Vascular invasion				0.85
Yes	1 (2.2)	4 (1.3)	2 (1.1)	
No	44 (97.8)	301 (98.7)	173 (98.9)	
Lymphatic invasion				0.39
Yes	1 (2.2)	24 (7.9)	13 (7.4)	
No	44 (97.8)	281 (92.1)	162 (92.6)	
Perineural infiltration				0.12
Yes	3 (6.7)	57 (18.7)	34 (19.4)	
No	42 (93.3)	248 (81.3)	141 (80.6)	
Grade of differentiation				0.92
I	5 (11.1)	43 (14.1)	26 (14.9)	
II	34 (75.6)	227 (74.4)	132 (75.4)	
III	6 (13.3)	35 (11.5)	17 (9.7)	

### Disease recurrence

Overall, 67 (12.8%) patients presented with local disease recurrence after mean duration of 19.3 ± 17.2 months. The group distribution was 11.1%, 11.5% and 15.4%, respectively (*p* = 0.43). Furthermore, 24 (4.6%) presented with secondary cervical lymph node metastasis (CLNM) after a mean duration of 19.7 ± 17.5 months. The group distribution was 6.7%, 4.3% and 4.6%, respectively (*p* = 0.77). Distant metastasis occurred in 46 (8.8%) cases, after a mean duration of 20.2 ± 17.9 months. Here, the group distribution was 8.9%, 9.2% and 8.0%, respectively (*p* = 0.91).

### Survival analysis

**Fig. 2:**
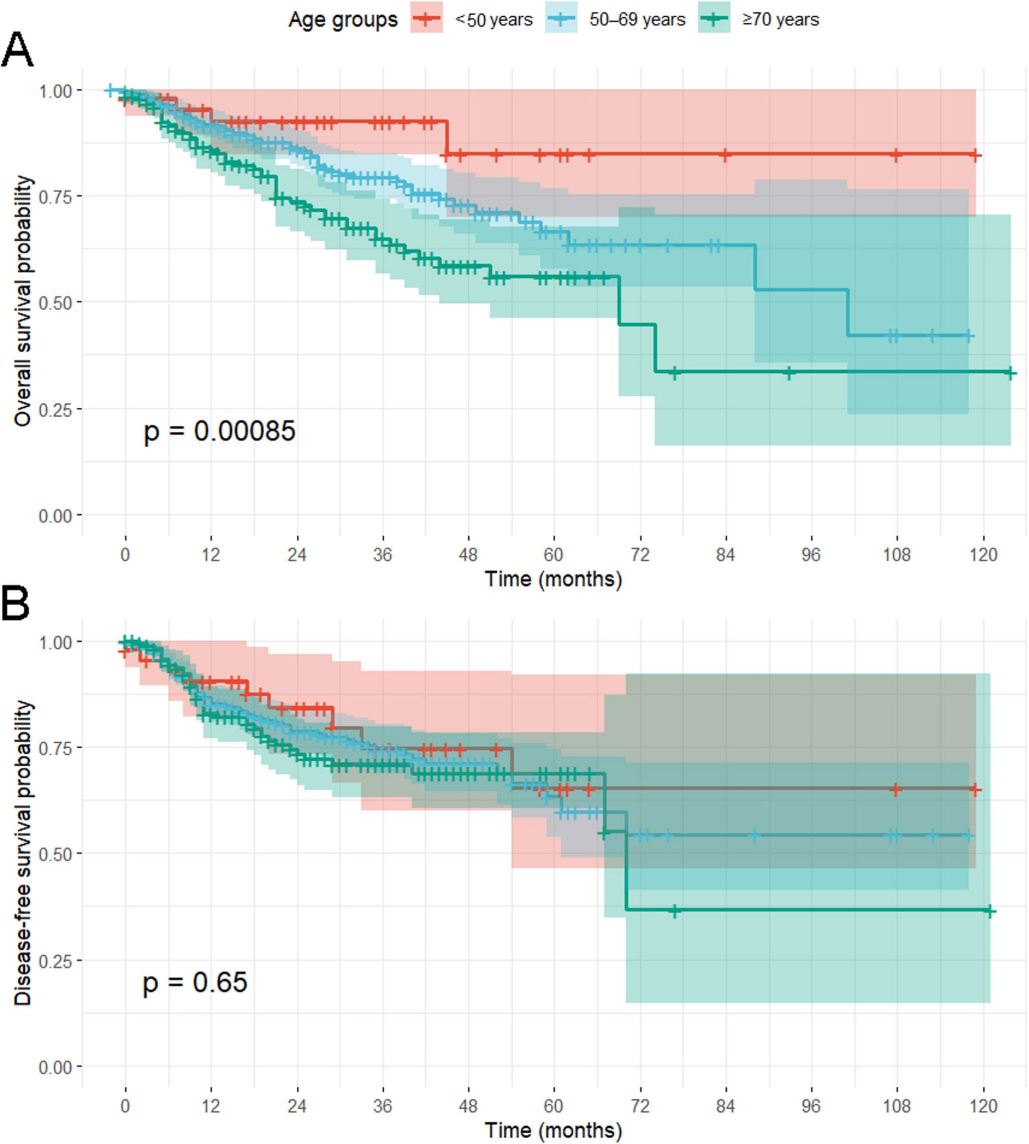
Kaplan Meier survival curves presenting the overall survival (**A**) and disease-free survival (**B**) in relation to the age groups

The 3-year OS and DFS were 75.6% and 73.3%, respectively. Survival differences among the age groups were statistically significant in relation to the OS (92.5% vs. 79.4% vs. 64.7%, *p* < 0.01) but not in relation to the DFS (74.6% vs. 74.3% vs. 70.9%, *p* = 0.65), as shown in Fig. 2. To assess how age behaves as a prognostic factor, we first assessed whether its effect on survival was linear or non-linear using spline functions. Since spline testing revealed no deviation from linearity, age was modeled continuously (per 10 years) in the multivariable Cox regression analyses, as presented in Table 2. When modeling age as a continuous variable with group-specific slopes, no significant effect of age was found in younger or middle-aged patients. In contrast, among elderly patients ≥ 70 years, each additional year of age was associated with a 6% increase in mortality risk (HR per 10 years: 1.70, 95%CI: 1.09–2.65, *p* = 0.02). In the competing-risk regression model, age was not associated with recurrence incidence (sHR: 0.99, 95% CI: 0.98–1.01, *p* = 0.78), whereas tumor-related factors such as nodal positivity, perineural infiltration and higher grades of differentiation remained significant predictors.

**Table 2:** Multivariable cox regression analyses for overall survival and disease-free survival, including hazard-ratios and 95% confidence intervals

	OS			DFS		
	HR	95% CI	*p*-value	sHR	95% CI	*p*-value
Age (10-fold)	1.56	1.29–1.90	< 0.01	0.99	0.98–1.01	0.78
Smoking	1.09	0.73–1.63	0.69	1.00	0.68–1.50	0.96
Sex	1.08	0-73-1.60	0.172	1.02	0.68–1.51	0.93
pT-Stage						
pT1	1 (Ref)	-	-	1 (Ref)	-	-
pT2	2.41	0.76–10.21	0.11	1.44	0.89–2.34	0.14
pT3	3.28	0.70-15.42	0.13	1.43	0.72–2.86	0.31
pT4a	4.52	0.99–20.60	0.05	1.48	0.86–2.55	0.16
pN+	1.73	0.77–3.90	0.18	1.86	1.14–3.05	0.01
ECS	2.24	1.32–3.81	< 0.01	1.48	0.82–6.48	0.79
Vascular invasion	2.54	0.60-10.72	0.21	1.01	0.89–1.12	0.79
Lymphativ invasion	1.38	0.77–2.48	0.28	1.02	0.87–1.18	0.71
Perineural infiltration	1.78	1.04–3.06	0.04	1.95	1.18–3.21	< 0.01
Grade of differentiation						
I	1 (Ref)	-	-	1 (Ref)	-	-
II	1.74	0.84–3.58	0.13	2.51	1.03–6.12	0.04
III	3.35	1.45–7.74	< 0.01	4.08	1.51–10.95	< 0.01

## Discussion

In this study, we demonstrated that within our patient cohort, which was intentionally not stratified for clinical or histopathological risk factors to reflect a natural cohort, age showed a multimodal distribution with three distinct groups. Younger patients were more often affected by tongue OSCC, whereas non-smoking status was more common among elderly patients, and male sex was most frequent in the middle-aged group. While T- and N-stage did not differ significantly between groups, younger patients showed a markedly higher ECS/pN+ ratio. Comparable patterns have been reported in the literature. Abdulla et al. found tongue and buccal mucosa to predominate in younger Indian patients (< 45 years), who also showed higher tobacco consumption, while elderly patients were less likely to have a smoking history, consistent with our results [15]. Sundar et al. reported a higher proportion of female patients and more frequent nodal positivity among the elderly, partially aligning with our findings, although our difference in nodal status did not reach significance [16]. Interestingly, the elevated ECS/pN+ ratio observed in our young subgroup has not been described elsewhere, to the best of our knowledge. Troeltzsch et al. also noted tongue as the predominant site in younger patients, whereas risk factor exposure was far less common in the very young, though interpretation was limited by a small sample size [17].

An interesting observation in our cohort was that, among younger patients, the correlation between secondary CLNM and distant metastasis was markedly stronger (*r* = 0.54, *p* < 0.01). A recent systematic review assessing the relation between patient age and different outcome measures identified young age as an independent prognostic factor for distant metastasis [18]. However, it is important to note that this observation was also for oropharyngeal SCC and thus not directly comparable to exclusively oral cavity carcinoma. Ding et al. demonstrated that lymph node ratio correlated with distant-metastasis-free survival, but this referred to primary nodal disease and was not stratified by age, leaving its relevance for younger patients uncertain [19]. However, it underlines the general relation between the extent of regional disease and distant metastasis. Assessing two different prediction models for distant metastasis, Lu et al. had similar findings [20]. The authors compared two models, incorporating either no or present initial CLNM (pN0 versus pN+), showing that locoregional recurrence predicted poorer distant-metastasis-free survival, yet without age-specific analyses. One study reported association for younger age in relation to metachronous carcinoma [21]. Here, younger age (< 65 years) was linked to an increased risk of metachronous esophageal cancer in hypopharyngeal SCC. Nevertheless, this association was not found in OSCC in their study, highlighting the difference among both entities.

Another main finding in our study was that age was significantly associated with an increased risk of death for overall survival, but not for disease-free survival. Importantly, the absence of an age effect on recurrence persisted after accounting for death without prior recurrence as a competing event in Fine–Gray regression models. This may indicate that the observed age-related disadvantage in overall survival is unlikely to be driven by differences in recurrence patterns or intrinsic tumor aggressiveness. Notably, established tumor-related parameters remained robust predictors of recurrence incidence. These results reinforce that recurrence risk in OSCC is primarily driven by biological tumor characteristics rather than patient age.

Goldenberg et al., analyzing a large SEER cohort including nearly 20,000 head and neck cacner patients with propensity score matching for clinical and histological variables, also reported inferior OS in older patients, consistent with our results [22]. In contrast, Malik et al. observed no significant difference in OS or DFS in their study [23]. Here, the authors only included patients above 65 years and dichotomized two study groups at 70 years, thus comparing only elderly patients without including younger ages. Furthermore, Oh et al. highlighted a survival advantage in young and middle-aged female patients, particularly in relation to DFS, suggesting possible sex-specific effects. In their multicenter analysis of 3,379 cases, patients were stratified by age and sex, and it was shown that especially young and middle-aged females benefitted in terms of DFS, even after stratification by smoking status, when compared to male patients [24]. A systematic review by Lenze et al. on tongue OSCC confirmed the heterogeneity of the literature, with only a minority of studies demonstrating higher recurrence rates in younger patients [25]. Specifically, 5-year disease recurrence rates varied between 30 and 72% for young patients and 42–81% for elderly patients. Taken together, these findings suggest that while age is consistently linked to OS, its role in predicting DFS remains less clear and may depend on patient subgroups, tumor sites, or treatment patterns.

This study has several limitations that should be acknowledged. First, its retrospective single-center design carries a certain risk of selection bias and may limit the generalizability of the findings to other populations. Given its explorative character, the study should primarily be regarded as hypothesis-generating, underscoring the need for validation in larger, prospective multicenter cohorts. Furthermore, an important limitation of this study is the exclusion of patients who did not receive guideline-recommended adjuvant therapy. This decision was made to minimize confounding by treatment heterogeneity, as undertreatment is strongly associated with inferior survival. However, this approach may have resulted in the selection of a biologically fitter subgroup of elderly individuals who were able to undergo standard therapy. Consequently, the observed association between age and overall survival may underestimate the true impact of age in an unselected population, and the generalizability of our findings to patients who are not eligible for standard treatment is limited. The distribution of patients across the age groups was uneven, with a relatively small number of younger patients, which reduces statistical power and makes subgroup analyses, particularly the correlation analyses, more vulnerable to random effects. Therefore, due to the limited number of events, these findings should be considered exploratory. However, in contrast to many previous studies with small sample sizes or arbitrary age cut-offs, a major strength of our work lies in the data-driven approach to age stratification, allowing for a more nuanced assessment of age as an independent prognostic factor and its relationship with different recurrence patterns.

## Conclusions

In this larger single-center cohort of surgically treated OSCC patients, increasing age was independently associated with worse overall survival but showed no association with disease-free survival, even after accounting for competing mortality. These findings suggest that the age-related survival disadvantage is unlikely to be driven by differences in tumor recurrence patterns and may instead reflect non-oncologic factors. Established tumor-related parameters remained the primary determinants of recurrence incidence. Taken together, our results support the interpretation that chronological age functions predominantly as a determinant of overall mortality rather than as a marker of distinct tumor aggressiveness in OSCC. Prospective studies incorporating comorbidity and functional status measures are warranted to further clarify the mechanisms underlying age-related survival differences.

## Data Availability

The author confirms that all data generated or analyzed during this study are included in this published article.
